# Assessment of Preclinical Antioxidative and Anti‐Inflammatory Activities of *Cornus macrophylla* Wall. Bark

**DOI:** 10.1002/fsn3.70620

**Published:** 2025-07-16

**Authors:** Ali Khan, Aini Pervaiz, Muhammad Saeed Jan, Bushra Ansari, Imad Ahmad, Syed Muhammad Mukarram Shah, Abdur Rauf, Ahood Khalid, Anees Ahmed Khalil, Hassan A. Hemeg, Yahya S. Al‐Awthan, Omar S. Bahattab, Mohammed Mansour Quradha

**Affiliations:** ^1^ Professional Institute of Health Sciences Mardan KP Pakistan; ^2^ Department of Pharmacy Bacha Khan University Charsadda KP Pakistan; ^3^ Department of Pharmacy University of Swabi Swabi KP Pakistan; ^4^ Department of Chemistry University of Swabi Khyber Pakhtunkhwa Pakistan; ^5^ University Institute of Diet and Nutritional Sciences, Allied Health Sciences The University of Lahore Pakistan; ^6^ Department of Clinical Laboratory Sciences College of Applied Medical Sciences Saudi Arabia; ^7^ Department of Biology, Science University of Tabuk Tabuk Saudi Arabia; ^8^ Biodiversity Genomics Unit, Science University of Tabuk Tabuk Saudi Arabia; ^9^ College of Education Seiyun University Yemen; ^10^ Pharmacy Department, Medical Sciences Aljanad University for Science and Technology Taiz Yemen

**Keywords:** anti‐inflammatory, lipid peroxidation, neuroprotective, spectrophotometry

## Abstract

*Cornus macrophylla* Wall. Bark has been traditionally utilized for its therapeutic properties; however, limited scientific evidence is available that supports its role as having anti‐inflammatory and neuroprotective properties. Exploring the neuroprotective and anti‐inflammatory properties of *C. macrophylla* Wall. Bark may help in validating the therapeutic properties, presenting insights into its use as a potent natural component for modulating inflammation and oxidative stress‐related neurological ailments. In this study, crude extract along with different fractions of *C. macrophylla* Wall. Bark have been investigated for anti‐inflammatory and neuroprotective effects. In the in vivo anti‐inflammatory assay, the ethyl acetate (EA) fraction showed activity of 68.3%, 79.7%, and 83.7% at dosages of 50, 100, and 200 mg/kg B.W. (Body weight) of all the mice decreased when treated with LPS while it increased when treated with aspirin, crude extract, dichloromethane (DCM), and EA, respectively. Rectal temperature increased when treated with lipopolysaccharide (LPS) and gradually decreased with aspirin, crude, DCM, and EA fraction. Moreover, locomotor activity was weak when treated with LPS and enhanced when treated with aspirin, crude, DCM, and EA, respectively. Neuroprotective ex vivo results showed that the EA fraction decreased oxidative stress by restoring the glutathione (GSH) level by 21.10, 18.20, and 14.04 μM/mg proteins at doses of 100, 200, and 300 mg/kg body weight, respectively. The EA fraction decreased oxidative stress by restoring the superoxide dismutase (SOD) level by 0.17, 0.15, and 0.13 units/mg proteins at doses of 100, 200, and 300 mg/kg BW, respectively. The EA fraction decreased oxidative stress by restoring the malondialdehyde (MDA) level by 0.17, 0.15, and 0.13 units/mg proteins at dosages of 100, 200, and 300 mg/kg body weight, respectively. The EA fraction markedly increases the level of catalase (CAT) by 46.26, 41.12, and 39.37 units/mg proteins at doses of 100, 200, and 300 mg/kg body weight, respectively. In vivo results validated the in vitro results of different fractions of *C. macrophylla* .

## Graphical abstract

*C. macrophylla* , belonging to the Cornaceae family, exhibits significant anti‐inflammatory and neuroprotective properties. The ethyl acetate fraction showed the highest efficacy in reducing oxidative stress, restoring antioxidant levels (GSH, SOD, TBARS, and CAT), and improving locomotor activity in LPS‐treated mice, validating its therapeutic potential.

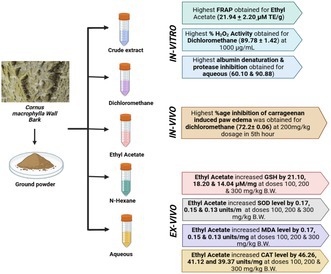

## 1 Introduction

*Cornus macrophylla* Wall. (family Cornaceae), also known by its indigenous name “large leaf dogwood”, has been widely consumed as a potent therapy for treating various ailments. Moreover, *C. macrophylla* is known among folk practitioners as diuretic, analgesic, and for preserving foods. However, the fruits of *C. macrophylla* are being used as a remedy against varied diseases such as allergies, malaria, inflammation, cancer, diabetes, and oxidative stress (Noshiro and Baas 2000; Shah et al. 2015).

Inflammation is a degenerative process resulting from the accumulation of low‐molecular‐weight catabolic entities generated from damaged cells, causing alteration of osmotic pressure. Swelling during inflammation occurs due to elevated osmotic pressure, which leads to the exudation of fluid from the cell into the surrounding tissue, with or without liberation of heat, depending upon the metabolic activity at the inflamed area. Numerous pathological conditions are linked to chronic inflammation, such as cancer, soreness, redness, increased tissue temperature, and organ failure (Pan et al. 2014). Neuro‐inflammation is a syndrome that occurs through the hallmarks associated with the loss of neuronal structure and function in the central nervous system, resulting from a viral insult, but not limited to it. Neurodegeneration is profusely noticed in old‐aged people, resulting in different neurological diseases. Parkinson's disease, multiple sclerosis, and Alzheimer's disease are caused due to neurodegeneration and pose detrimental effects on mental and physical functionalities. It is still important to classify the causative agents of neurodegeneration. The inflammatory mechanism, however, has been found to be among the main reasons leading to multiple neurodegenerative pathways that are associated with depression. In the pathophysiology of neurodegeneration, pro‐inflammatory cytokines are found to play intriguing roles and therefore, are considered essential in managing neurodegenerative disorders.

Clinically, administration of carrageenan manifests weight loss, diarrhea, and visible blood in feces due to intestinal inflammation and mucosal injury induced by carrageenan exposure (Estakhr and Javdan 2011). Histamine, serotonin, and bradykinin are the first detectable mediators in the early phase of carrageenan‐induced inflammation; prostaglandins are involved in the increased vascular permeability and are detectable in the late phase of inflammation. Local and/or systemic inflammation is associated with enhanced levels of the pro‐inflammatory cytokines TNF‐*α*, IL‐1, and IL‐6 (Wu et al. 2022). Previous studies have highlighted the role of superoxide dismutases (SODs), metalloenzymes present across various prokaryotes and eukaryotes, as major components of cellular antioxidant defense mechanisms (Landis and Tower 2005). These enzymes aid the conversion of •O_2_^−^ (superoxide anion free radical) to H_2_O_2_ (hydrogen peroxide) and O_2_ (molecular oxygen). Afterward, hydrogen peroxide in the presence of CAT (catalase), GPx (glutathione peroxidase), and/or Trx (thioredoxin)‐dependent Prx (peroxiredoxin) is reduced to water (Lesser 2005).

Catalase enzyme is an oxidoreductase enzyme as it plays a crucial role in quenching the reactive oxygen species (ROS), that is, hydrogen peroxide, often produced as a by‐product of aerobic respiration (Kaushal et al. 2018). The basic mechanism of the working of this enzyme involves the breakdown and subsequent breakdown of the reactive oxygen species, that is, hydrogen peroxide (H_2_O_2_), into oxygen and water, thus relieving the oxidative stress caused by this substrate as depicted in the following reaction (Vasiliev et al. 2001). Lipid peroxidation is one of the main consequences of oxidative stress, a process in which reactive oxygen species degrade polyunsaturated fatty acids in cell membranes. This degradation forms different toxic byproducts, among which malondialdehyde (MDA) is a well‐recognized marker of inflammation. Increased content of MDA indicates the extent of oxidative damage, contributes to cellular dysfunction, and contributes to the progression of inflammation‐related disorders. Hence, in biological systems, MDA is considered an authentic indicator of oxidative stress and inflammatory conditions (Ayala et al. 2014). Malondialdehyde (MDA) is a solid compound (melting point, 72°C–74°C) that is soluble in water, methanol, ethanol, and moderately soluble in methylene chloride, while insoluble in diethyl ether (Wencel‐Delord et al. 2001), with a *pK*_*a*_ value of 4.46 (Tsikas 2017). MDA level is commonly known as a marker of oxidative stress and the antioxidant status in cancerous patients (Gaweł et al. 2004). MDA is the prototype of the so‐called thiobarbituric acid reactive substances (TBARS). Glutathione reductase (GR) plays an essential central role in cell defense against reactive oxygen metabolites by efficiently maintaining the cellular reduced GSH pool through catalyzing the reduction of GSSG to GSH with the accompanying oxidation of NADPH (Anjum et al. 2010; Trivedi et al. 2013).

It is evident from the literature that medicinal plants and natural products have potential in curtailing inflammation and achieving optimal health (Calder et al. 2017; Pan et al. 2010). Herbal and medicinal plants provide an alternative and safe approach to potentially address chronic inflammatory conditions that are directly linked to several ailments such as diabetes, cardiovascular complications, and other metabolic disorders (Agarwal and Shanmugam 2022; Tasneem et al. 2019). It is the need of the time to investigate the potential of medicinal plants and their constituents in managing inflammation. Overall health and wellness can be achieved through dietary modifications and interventions. The present study aimed to highlight the effectiveness of *C. macrophylla* Wall. bark extracts in modulating inflammation, hence providing insights for their integration as dietary intervention and therapeutic strategies. Keeping in view the medicinal properties of *C. macrophylla* Wall. bark, this study was designed to evaluate the crude extract along with different fractions of *C. macrophylla* Wall. bark for in vivo anti‐inflammatory potential via carrageenan‐induced paw edema in mice. The experimental fractions were further subjected to lipo‐polysaccharide (LPS) induced neuro‐protective effect by performing various ex vivo activities.

## 2 Materials and Methods

### 2.1 Plant Materials

Bark of *C. macrophylla* Wall. was procured from the local market of Malakand District, KPK (Khyber Pakhtunkhwa), Pakistan in April. Prof. Dr. Nisar Ahmad (Department of Botany), University of Malakand, authenticated the plant's bark, and the specimen was stored in the herbarium of the department against voucher # H.B.UOM‐102.

### 2.2 Extraction and Fractionation

The bark (4.5 kg) was washed and shade‐dried, followed by grinding in a commercial grinder to collect respective bark powder. The bark powder was macerated in methanol for 15 days with continuous stirring. After maceration, the crude extract was initially filtered using muslin cloth to separate coarse solid particles, followed by filtration through filter paper. The filtered extract was concentrated at 40°C by the application of a rotary evaporator. After concentration, the final weight of the extract was 510 g with a yield of 11.3% which was measured using the standard formula ((Weight of extract after concentration/Weight of dried plant) * 100). For pharmacological analysis, out of 510, 260 g of crude methanolic extract was separated and stored till further analysis. Meanwhile, the remaining 250 g were subjected to fractionation through n‐hexane (NH), ethyl acetate (EA), and dichloromethane (DCM). Initially, the methanolic extract (250 g) was mixed with distilled water (500 mL), which was later mixed with n‐hexane (500 mL) for the preparation of a reaction mixture in a separating funnel, which was mixed thoroughly. The resultant reaction mixture was held for 5 min to ensure the formation of two immiscible layers. To obtain the n‐hexane fraction, the top layer was removed and concentrated using a rotary concentrator. To ensure effective removal of the n‐hexane fraction, this procedure was performed thrice. To collect the DCM fraction, the DCM was mixed with the aqueous layer of the crude methanolic extract solution. Again, the solution was held for a few minutes to ensure the formation of two immiscible layers of dissimilar solvents. To obtain the DCM fraction, the bottom layer was concentrated in a rotary concentrator. To ensure effective removal of the DCM fraction, this procedure was performed thrice. Similarly, in a separating funnel, ethyl acetate was added to the top aqueous layer and mixed thoroughly. Finally, this solution was again held for a few minutes to ensure the formation of two immiscible layers. The ethyl acetate fraction was removed from the solution and concentrated using a rotary concentrator. To ensure effective removal of the EA fraction, this procedure was performed thrice. The fraction left at the end of the procedure was an aqueous fraction (Ngo et al. 2018; Sadiq et al. 2019).

### 2.3 In Vitro Antioxidant Activity

#### 2.3.1 Ferric Reducing Antioxidant Power (FRAP) Assay

To evaluate the in vitro antioxidant potential of extracts and fractions, in this study the FRAP assay was conducted by following the protocols adopted by Du et al. (2019). Initially, 10 times diluted extract (1 mL) was added to PBS (0.2 mL) and potassium ferricyanide (1.5 mL: 0.3%), followed by incubation for 20 min at 50°C. Afterward, 1 mL trichloroacetic acid (10%) was mixed with the incubated solution, followed by centrifugation (3000 rpm) for 10 min. Later, the resultant supernatant (2 mL) was mixed with distilled water (3 mL) and 0.5 mL of ferric trichloride (3%). In the end, the absorbance of the final mixture was noted at 700 nm. The results were presented as μM TE/g fw.

#### 2.3.2 Hydrogen Peroxide Free Radical Assay

To evaluate the in vitro antioxidant potential through hydrogen peroxide free radical assay of extracts and fractions, in this study, the protocols described by Ruch et al. (1989) were adopted. Purposely, hydrogen peroxide solution (2 mmol) was made in phosphate buffer (50 mmol) at 7.4 pH. 0.1 mL of extracts and fractions were mixed in phosphate buffer (50 mmol) to raise the final volume to 0.4 mL. Before checking the final absorbance with spectrophotometer at 230 nm, 0.6 mL of hydrogen peroxide was mixed in each test tube containing extracts and fractions, respectively. Following formula was used for determination of percent hydrogen peroxide scavenging activity;

Hydrogen peroxide scavenging activity=1–sample absorbance/control absorbance×100

### 2.4 In Vitro Anti‐Inflammatory Activity

#### 2.4.1 Inhibition of Albumin Denaturation

Inhibition of albumin denaturation method was used for assessing the anti‐inflammatory properties of extracts and fractions. For this purpose, 0.05 mL of extracts and fractions in varied concentrations (31.25–1000 mg mL^−1^) were mixed with 0.5 mL of albumin aqueous solution (5%). 1 N hydrochloric acid was added to the prepared solution for adjustment of pH to 6.3. The resultant solution was given a stay time of 20 min at 37°C and heated afterward for 3 min at 51°C. Later, the reaction solution was cooled, and phosphate buffer (2.5 mL) was mixed in it, followed by measurement of absorbance at 660 nm (Jan et al. 2020). The following formula was used for measuring the percent inhibition of protein denaturation;

%Inhibition=Absof control−Absof sample/Absof control×100

#### 2.4.2 Protease Inhibitory Assay

In this assay, each 2 mL of reaction mixture contained trypsin (0.06 mg mL^−1^), Tris‐HCl buffer (1 mL of 20 mM), and extracts/fractions (1 mL) at varied concentrations ranging from 31.25 to 1000 mg mL^−1^. This mixture was given a stay time of 5 min at 37°C, followed by the addition of casein (1 mL of 0.8%) before again incubating for 20 min and adding perchloric acid (2 mL of 70%). Finally, before measuring the absorbance at 217 nm, the cloudy suspension was subjected to centrifugation (3000 rpm) for 5 min (Jan et al. 2020). The following formula was used for measuring the percent inhibition of protease;

%Inhibition=Absof control−Absof sample/Absof control×100

### 2.5 Acute Toxicity Testing

For acute toxicity studies, BALB/C mice (male) were divided into five groups (*n* = 6). The first group was considered as control, while the other four groups were treated with different doses/concentrations of extract and its fractions. The animals were observed for 1–2 h for any gross effects, and the mortality of mice was checked after 24 h.

### 2.6 Assay for Carrageenan‐Induced Paw Edema

The crude extract of *C. macrophylla* Wall. bark and its several fractions as NH, DCM, EA, and AQ were screened for their anti‐inflammatory effect. Albino mice weighing 25–30 g of both sexes were selected. Six different groups were made of selected animals. Each group consisted of six mice (*n* = 6). For negative and positive controls, Group I and Group II were selected. At a 10 mL/kg body weight dose, normal saline was administered to Group I as a negative control, and at a 10 mg/kg body weight dose, diclofenac sodium was administered to Group II as a positive control group. To the remaining groups (IV, V, and VI), different extracts were administered at various doses (50, 100, and 200 on body weight), respectively. Carrageenan 1% was injected into the subplanter right‐hand paw after 30 min of above treatment to each mouse, and the anti‐inflammatory effect was measured with the help of a plethysmometer for 5 h at 0, 1, 2, 3, 4 h, and 5 h) after administration of carrageenan (Khan et al. 2009). The paw edema percent inhibition will be calculated using the standard formula as follows.

$$\%\text{inhibition}=\underline{A-B}\times 100\;A$$

Where an A is the negative control edema volume, while B represents tested group paw edema.

### 2.7 Assay for LPS‐Induced Inflammatory Activity

*C. macrophylla* bark crude extract and its fractions DCM and EA for the presence of neuroprotective effects were screened. Albino mice for this purpose, having an average weight of 25–30 g body weight were selected. The mice were divided into five groups, each having six mice. Control group I first received normal saline for 14 days orally once daily. The second group II was treated with normal saline orally for 14 days, followed by intraperitoneal LPS (Beijing Solarbio Technology Co. Ltd. CAS No: 111C039) administration at 100 μg/kg body weight. The third group III of mice was treated with aspirin at a dose of 200 mg/kg body weight orally for 14 days, followed by LPS administration intraperitoneally at a dose of 100 μg/kg body. IV, V, and VI groups of mice were treated with above‐mentioned three extracts of *C. macrophylla* bark orally at a dose of 100, 200, and 300 mg/kg body weight, followed by LPS intraperitoneally at a dose of 100 μg/kg body weight. After 14 days of treatment, several behavioral assessments were carried out, like body weight, rectal temperature, and locomotor activity (Prakash et al. 2017).

### 2.8 Dissection and Homogenization

The mice were sacrificed on the 15th day with mild anesthesia. Their brains were removed, and the forebrain was dissected out after the completion of the behavioral assessment posttreatment period. Brains dissected were placed on ice and rinsed with isotonic saline for the removal of blood. A 0.1 M phosphate buffer was added to make the resultant homogenate (Prakash et al. 2017).

### 2.9 Estimation of Glutathione Reductase

For determining the activity of glutathione reductase, a reaction mixture of NADPH, EDTA, oxidized glutathione, and phosphate buffer was formed by using distilled water. Tissue homogenate was mixed with the reaction mixture, and absorbance was measured at 340 nm for 2 min at each 30 s interval. Results were presented as mol NADPH oxidized min^−1^ mg^−1^ protein (Davidson and Hird 1964).

### 2.10 Estimation of Superoxide Dismutase

For determining the activity of glutathione reductase, the absorbance of the reaction mixture comprising epinephrine and carbonate buffer was noted at 480 nm for 2 min at a regular interval of 15 s (Misra and Fridovich 1976).

### 2.11 Assay for TBARS (Thio‐Barbituric Acid Reactive Substances)‐Malonaldehyde Level

In order to assess the lipid peroxidation, the reaction of TBARS was measured using an indirect determination method. Homogenates were mixed with trichloroacetic acid along with TBA and HCl (0.25 M) in test tubes. The solutions were mixed vigorously and placed in an ice bath to lower the temperature. Afterward, the solution was subjected to centrifugation for 10 min. The top layer was separated, and absorbance was measured at 532 nm (Ohkawa et al. 1979).

### 2.12 Determination of Catalase Level

The measurement of catalase level was assessed by the procedure of Aebi (1984). The reaction mixture consisted of phosphate buffer, tissue homogenate, and hydrogen peroxide. The absorbance (240 nm) was noted for 30 s at intervals of 15 s.

### 2.13 Determination of Body Weight

Digital weighing balance was used for determination of body weights, before and after the administration of extracts/fractions and drug control.

### 2.14 Determination of Rectal Temperature

A thermostat probe was used for measuring rectal temperature. For this purpose, the probe (1.3 cm) was inserted into the rectum and taped to the base of the tail.

### 2.15 Assessing Locomotor Activity

Actophotometer digital apparatus was used to monitor locomotor activity. The apparatus was placed in a light and sound‐attenuated, darkened testing room. There was a digital counter that presented any pause of the beam on the x‐axis or y‐axis and created a subsequent electric impulse. On Day 14th, activities were performed on animals followed by LPS administration, and at counts per 5 min, values were expressed respectively (Reddy and Kulkarni 1998).

## 3 Results

### 3.1 In Vitro Antioxidant Assay

The in vitro antioxidant assay investigated the free radical scavenging potential of methanolic extract of *C. macrophylla* along with its prepared fractions. Results revealed that DCM and EA fractions possessed the most potential FRAP activities, that is, 21.65 and 21.94 μM TE/g, respectively. The other fractions, like crude, n‐Hexane, and aqueous, also displayed good to moderate activity, causing 8.46, 11.24, and 7.59 μM TE/g FRAP inhibition, as shown in Table 1.

**TABLE 1:** The ferric reducing antioxidant power (FRAP) assay of crude extract and different fractions of *C. macrophylla* .

Item	FRAP (μM TE/g)
Dichloromethane	21.65 ± 3.14
Ethyl acetate	21.94 ± 2.20
Crude	8.46 ± 0.60
*n*‐Hexane	11.24 ± 1.56
Aqueous	7.59 ± 0.76
*p*	< 0.001

#### 3.1.1 Hydrogen Peroxide Free Radical Assay

In the H_2_O_2_ free radicals scavenging activity, DCM and EA again showed the highest activities causing 89.78% ± 1.42%, 83.22% ± 0.78%, 75.56% ± 0.42%, 70.34% ± 0.44%, 66.42% ± 0.64% and 87.77% ± 0.73%, 82.22% ± 1.44%, 77.54% ± 0.52%, 73.49% ± 0.45%, 65.10% ± 0.34% inhibitions, respectively at concentrations ranging from 1000 to 62.5 μg/mL with IC_50_ values of 16.38 and 12.68 μg/mL. Similarly, the other fractions, like crude, hexane, and aqueous, displayed 85.15% ± 0.72%, 76.62% ± 0.66%, and 71.51% ± 0.77% inhibition at 1 mg/mL concentration (Table 2). The calculated IC_50_ values were 13.56, 31.54, and 93.41 μg/mL, respectively. Ascorbic acid inhibition was 96.54% ± 0.66%, 91.46% ± 0.34%, 86.62% ± 0.46%, 82.80% ± 0.84%, and 77.37% ± 0.78% at the same tested concentration, with an IC_50_ of 7.94 μg/mL.

**TABLE 2:** H_2_O_2_ inhibitory assay of the crude extract and different fractions of *C. macrophylla* .

Samples names	Concentration (μg/mL)	% H_2_O_2_ activity	IC_50_ (μg/mL)
Crude	1000 500 250 125 62.5	85.15 ± 0.72*** 81.70 ± 0.40*** 75.64 ± 0.62*** 71.22 ± 0.20*** 64.34 ± 0.94***	13.56
*n*‐Hexane	1000 500 250 125 62.5	76.62 ± 0.66*** 64.34 ± 0.94*** 70.38 ± 0.12*** 63.16 ± 0.44*** 57.42 ± 0.54***	31.54
Dichloromethane	1000 500 250 125 62.5	89.78 ± 1.42*** 83.22 ± 0.78*** 75.56 ± 0.42*** 70.34 ± 0.44*** 66.42 ± 0.64***	16.38
Ethyl acetate	1000 500 250 125 62.5	87.77 ± 0.73*** 82.22 ± 1.44*** 77.54 ± 0.52*** 73.49 ± 0.45*** 65.10 ± 0.34***	12.68
Aqueous	1000 500 250 125 62.5	71.51 ± 0.77*** 66.34 ± 0.92*** 58.57 ± 0.51*** 52.22 ± 0.34*** 46.79 ± 0.47***	93.41
Ascorbic acid	1000 500 250 125 62.5	96.54 ± 0.66 91.46 ± 0.34 86.62 ± 0.46 82.80 ± 0.84 77.37 ± 0.78	7.94

*Note:* Data are represented as mean ± S.E.M; *n* = 3, *represents level of significance, like: ***p < 0.001.

### 3.2 In Vitro Anti‐Inflammatory Activity

#### 3.2.1 Inhibition of Albumin Denaturation and Protease

Among our tested samples, it was noticed that EA fraction showed good albumin denaturation (IC_50_: 22.15 μg) and protease inhibition (IC_50_: 14.20 μg) potentials. The other fractions showed moderate activity in these assays (Table 3).

**TABLE 3:** In vitro albumin denaturation and protease inhibition activities of *C. macrophylla* .

Tested fractions	Albumin denaturation IC_50_ (μg)	Protease inhibition IC_50_ (μg)
Crude	27.10	21.88
*n*‐Hexane	45.40	40.32
Dichloromethane	25.30	15.80
Ethyl acetate	22.15	14.20
Aqueous	60.10	90.88
Diclofenac sodium	20.10	13.67

*Note:* The calculated IC_50_ value was μg. Data are represented as mean ± S.E.M; *n* = 3.

### 3.3 Acute Toxicity

The results for acute toxicity displayed no associated mortalities in mice administered a single dosage of 3000 mg/kg of any of the investigated crude extracts. 3000 mg/kg is the lethal dose (LD_50_) for crude drug extract. No signs of illness or behavioral changes were noticed in mice during this study trial (Table 4).

**TABLE 4:** Acute toxicity result of crude drug extract of *C. macrophylla* bark.

Group	Animals	Tested crude drug toxicity (mg/kg b. wt)
1	5	500
2	5	1000
3	5	1500
4	5	2000
5	5	3000

### 3.4 In Vivo Anti‐Inflammatory Activity

In carrageenan induced paw edema assay, the EA fraction of *Cornus mass L*. demonstrated excellent activity against carrageenan induced inflammation at dose of 200 mg/kg which is the highest dose of that extract, and reached its maximum value at the 4th hour, remaining significant (***p < 0.001) until the 5th hour of sample administration. At the 4th hour, the EA fraction showed an activity of 83.7 ± 0.03 at a dose of 200 mg/kg body weight, while the standard drug, that is, diclofenac sodium, showed marked activity of 80.8 ± 0.80 and remained significant up to the 5th hour. Similarly, the dichloro methane fraction also exhibited marked activity of 77.2 ± 0.08 at a dose of 200 mg/kg body weight at the 4th hour. The remaining fractions also exhibited good activity, but the EA fraction remained the most active fraction among all. The n‐hexane fraction (61.2 ± 0.22) is more active than the aqueous fraction, which had an activity of 57.9 ± 0.07 at a dose of 200 mg/kg body weight, as shown in Table 5.

**TABLE 5:** Percent inhibition of various extracts of *C. macrophylla* bark in carrageenan‐induced paw edema.

Test sample/drug	Dose mg/Kg	1st hour	2nd hour	3rd hour	4th hour	5th hour
Vehicle	10 ml	70.0 ± 0.03	71.5 ± 0.07	71.0 ± 0.03	71.2 ± 0.07	71.3 ± 0.03
Diclofenac sodium	10 mg	71.8 ± 0.80	74.2 ± 0.06	80.8 ± 0.80	77.7 ± 0.70	73.8 ± 0.04
Crude	50 100 200	42 ± 0.02^ns^ 51 ± 0.05^ns^ 60 ± 0.04***	56 ± 0.04^ns^ 57.6 ± 0.02^ns^ 62 ± 0.02***	58.75 ± 0.03^ns^ 63 ± 0.03^ns^ 65.8 ± 0.08***	54.4 ± 0.20^ns^ 51.6 ± 0.04^ns^ 54.4 ± 0.40***	51.6 ± 0.08^ns^ 51.6 ± 0.60^ns^ 55.8 ± 0.06***
EA	50 100 200	61.9 ± 0.20^ns^ 67.5 ± 0.30^ns^ 73.4 ± 0.40***	63.4 ± 0.40^ns^ 72.3 ± 0.30^ns^ 76.1 ± 0.50**	66.6 ± 0.20 ^ns^ 76.2 ± 0.20 ^ns^ 81.2 ± 0.40***	68.3 ± 0.15^ns^ 79.7 ± 0.31^ns^ 83.7 ± 0.03^ns^	59.4 ± 0.20^ns^ 62.7 ± 0.50^ns^ 68.3 ± 0.03**
DCM	50 100 200	58.7 ± 0.30^ns^ 60.9 ± 0.50^ns^ 68.4 ± 0.08***	58.8 ± 0.02^ns^ 66 ± 0.06^ns^ 72.6 ± 0.04***	63 ± 0.50^ns^ 70.8 ± 0.08^ns^ 75 ± 0.50***	57 ± 0.30^ns^ 73.8 ± 0.02^ns^ 77.2 ± 0.08***	52.2 ± 0.04^ns^ 67.2 ± 0.02^ns^ 72.2 ± 0.06***
NH	50 100 200	44 ± 0.08^ns^ 51 ± 0.11^ns^ 57.5 ± 0.50***	46 ± 0.06^ns^ 53 ± 0.30^ns^ 58.8 ± 0.06***	51.6 ± 0.04^ns^ 54.1 ± 0.30^ns^ 61.2 ± 0.22***	42.2 ± 0.06 ^ns^ 43.4 ± 0.08 ^ns^ 51.6 ± 0.02***	40 ± 0.02^ns^ 41.1 ± 0.11^ns^ 47.7 ± 0.07***
AQ	50 100 200	39.3 ± 0.11^ns^ 45 ± 0.30^ns^ 53.7 ± 0.07***	44.2 ± 0.02^ns^ 47.3 ± 0.50^ns^ 54.6 ± 0.08***	46.2 ± 0.02^ns^ 49.5 ± 0.30^ns^ 57.9 ± 0.07***	37.2 ± 0.08^ns^ 41.1 ± 0.50^ns^ 53.3 ± 0.30***	35.4 ± 0.04^ns^ 40 ± 0.06^ns^ 43.4 ± 0.08***

*Note:* ns: Non‐significant, Significant: *p < 0.05, **p < 0.01, ***p < 0.001.

#### 3.4.1 Effect of *C. macrophylla* Bark on LPS‐Induced Changes in Body Weight

**FIGURE 1:**
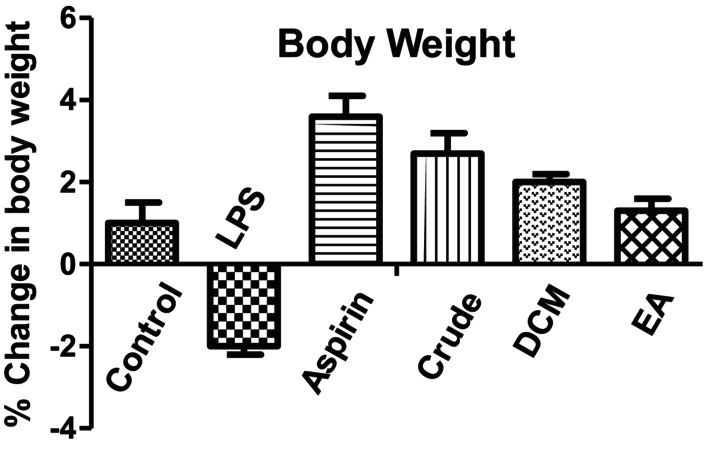
*C. macrophylla* bark, different extracts effects on weight alteration by LPS administration. Values are represented by mean ± SEM. *p* < 0.001 compared to control mice. *p* < 0.001 compared to LPS‐treated mice.

LPS‐treated mice at a dose of 100 μg kg^−1^ body weight momentously (*p* < 0.001) reduced the body weight in comparison to the control group. The mice treated group with aspirin at a dose of 200 mg/kg body weight showed increased significantly (*p* < 0.001) in body weight as compared to the LPS‐treated mice. Different *C. macrophylla* bark extracts, like crude extract, DCM, and ethyl acetate, showed a significant (*p* < 0.001) increase in body weight in comparison with the LPS‐treated mice (Figure 1).

#### 3.4.2 Effect of *C. macrophylla* Bark on LPS‐Induced Changes in Rectal Body Temperature

**FIGURE 2:**
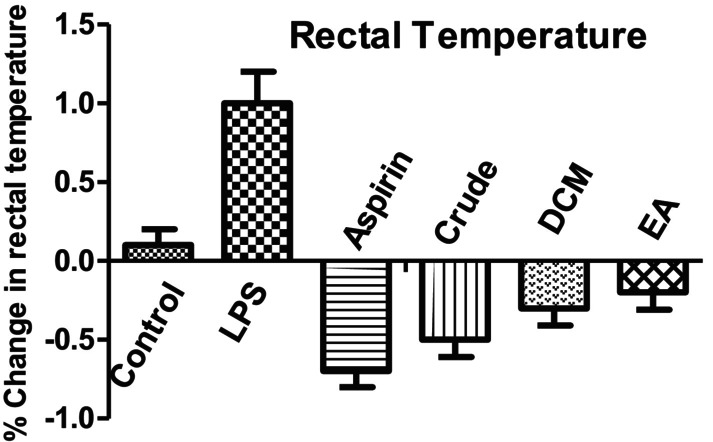
Effect of different extracts of *C. macrophylla* on the LPS‐induced variation in rectal temperature. Values are represented by mean ± SEM. *p* < 0.001 compared with control mice. *p* < 0.001 compared with LPS‐treated mice. *p* < 0.001 compared to aspirin‐treated mice.

LPS‐induced mice, when treated with aspirin at a dose of 200 mg/kg body weight, showed a significant reduction in rectal temperature as compared to the LPS‐treated group. Different *C. macrophylla* extract‐treated groups, like crude, DCM, and EA, revealed a reduction (*p* < 0.001) in rectal temperature when compared with the LPS‐treated mice (Figure 2).

#### 3.4.3 Effect of *C. macrophylla* Bark on LPS‐Induced Alteration in Locomotor Activity

**FIGURE 3:**
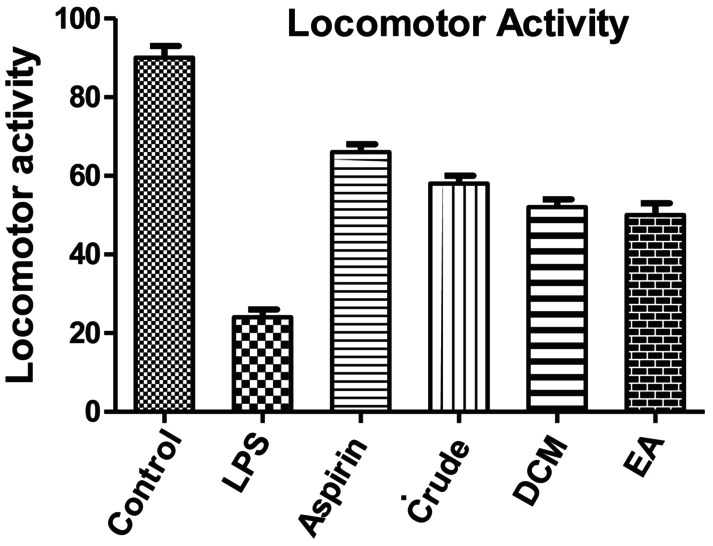
Effect of different extracts of *C. macrophylla* on LPS‐induced variation in locomotor activity using actophotometer. Values are represented by mean ± SEM, *p* < 0.001 when compared with control mice and *p* < 0.001 compared to the LPS‐treated mice.

LPS‐treated mice, when subjected to aspirin treatment (200 mg/kg body weight), the locomotor activity showed significant reduction as compared to the untreated control group. Pretreatment with different *C. macrophylla* extracts like crude, DCM, and EA momentously (*p* < 0.001) elevated the locomotor activity as compared to LPS‐treated mice (Figure 3).

### 3.5 Effect of *C. macrophylla* on Antioxidant Level

Antioxidant activities include the below‐mentioned ex vivo activities.

#### 3.5.1 Effect of *C. macrophylla* on LPS‐Induced Changes in GSH Level

LPS induction reduced the GSH level in mice, which was elevated on administration of aspirin as compared to LPS‐treated mice. Similarly, the mice treated with *C. macrophylla* in doses of 100, 200, and 300 mg/kg body weight also enhanced the GSH levels significantly (*p* < 0.001) as compared to LPS‐treated mice (Table 6).

**TABLE 6:** Effect of various fractions of *C. macrophylla* on LPS‐induced alterations in GSH levels.

Treatment		GSH (μmoles/mg protein)
Control		34.00 ± 0.60
LPS + aspirin		15.00 ± 0.92***
EA	(100 mg/kg)	21.10 ± 0.80***
	(200 mg/kg)	18.20 ± 0.62***
	(300 mg/kg)	14.04 ± 1.02***
CHF	(100 mg/kg)	27.08 ± 1.10n.s.
	(200 mg/kg)	25.40 ± 1.20*
	(300 mg/kg)	22.00 ± 1.10***
Crude	(100 mg/kg)	31.50 ± 1.20n.s.
	(200 mg/kg)	27.00 ± 1.40*
	(300 mg/kg)	24.00 ± 0.90***

*Note:* Values were shown as mean ± SEM (*n* = 5). One‐way ANOVA followed by Bonferroni posttest was applied, and data were represented as significant values as *p* values. n.s.; not significant and *p* > 0.05, *p < 0.05, **p < 0.01, ***p < 0.001.

#### 3.5.2 Effect of *C. macrophylla* on LPS‐Induced Alterations in SOD Level

Mice group treated with LPS significantly (*p* < 0.001) decreased the levels of superoxide dismutase when compared with the control group. Whereas the SOD levels were enhanced in the aspirin‐treated mice group. In the experimented groups, *C macrophylla* at doses of 100, 200, and 300 mg/kg body weight restored SOD level momentously (*p* < 0.001). However, the antioxidant effect showed at 300 mg/kg was much better than that of *C. macrophylla* (200 and 100 mg/kg) (Table 7).

**TABLE 7:** Effect of various fractions of *C. macrophylla* on LPS‐induced alterations in SOD levels.

Treatment		SOD (units/mg protein)
Control		0.28 ± 0.004
LPS + Aspirin		0.15 ± 0.008***
EA	(100 mg/kg)	0.17 ± 0.007**
	(200 mg/kg)	0.15 ± 0.006***
	(300 mg/kg)	0.13 ± 0.007***
CHF	(100 mg/kg)	0.19 ± 0.005*
	(200 mg/kg)	0.17 ± 0.008**
	(300 mg/kg)	0.15 ± 0.009***
Crude	(100 mg/kg)	0.20 ± 0.004n.s.
	(200 mg/kg)	0.18 ± 0.007**
	(300 mg/kg)	0.16 ± 0.009***

*Note:* Values were shown as mean ± SEM (*n* = 5). One‐way ANOVA followed by Bonferroni posttest was applied, and data was represented as significant values as *p* values. n.s; not significant and *p* > 0.05, *p < 0.05, **p < 0.01, ***p < 0.001.

#### 3.5.3 Effect of *C. macrophylla* on LPS‐Induced Alterations in MDA Level

The MDA levels in LPS‐treated mice were elevated compared to the control group. The MDA levels were reduced in the aspirin‐treated mice group as compared to the LPS‐treated mice group. Similarly, the MDA levels in pretreated *C. macrophylla* at doses of 100, 200, and 300 mg/kg body weight decreased significantly (*p* < 0.001) as compared to the LPS‐treated mice (Table 8).

**TABLE 8:** Effect of various fractions of *C. macrophylla* on LPS‐induced alterations in MDA levels.

Treatment		MDA (nmoles/mg protein)
Control		3.20 ± 0.08
LPS + Aspirin		2.40 ± 0.07***
EA	(100 mg/kg)	2.90 ± 0.07*
	(200 mg/kg)	2.42 ± 0.08***
	(300 mg/kg)	2.20 ± 0.08***
CHF	(100 mg/kg)	3.30 ± 0.09n.s.
	(200 mg/kg)	3.10 ± 0.09*
	(300 mg/kg)	2.50 ± 0.09***
Crude	(100 mg/kg)	3.20 ± 0.09n.s.
	(200 mg/kg)	3.10 ± 0.08*
	(300 mg/kg)	2.70 ± 0.08**

*Note:* Values were shown as mean ± SEM. (*n* = 5). One‐way ANOVA followed by Bonferroni posttest was applied, and data were represented as significant values as *p* values. n.s; not significant and *p* > 0.05, *p < 0.05, **p < 0.01, ***p < 0.001.

#### 3.5.4 Effect of *C. macrophylla* on LPS‐Induced Changes in CAT Level

CAT levels were diminished in the LPS‐treated mice group as compared to the control group of mice. Meanwhile, the aspirin‐treated mice group significantly (*p* < 0.001) enhanced the level of the CAT enzyme compared to LPS‐treated mice. Moreover, the level of CAT increased in the pretreated *C. macrophylla* (100, 200, and 300 mg/kg body weight) group as compared to LPS‐treated mice (Table 9).

**TABLE 9:** Effect of various fractions of *C. macrophylla* on LPS‐induced alterations in CAT levels.

Treatment		CAT (unit/mg protein)
Control		58.10 ± 0.92
LPS + Aspirin		38.10 ± 1.20***
EA	(100 mg/kg)	46.24 ± 0.82**
	(200 mg/kg)	41.12 ± 0.80***
	(300 mg/kg)	39.37 ± 0.93***
CHF	(100 mg/kg)	51.52 ± 1.52n.s.
	(200 mg/kg)	47.10 ± 1.20*
	(300 mg/kg)	42.22 ± 1.20***
Crude	(100 mg/kg)	54.60 ± 1.60n.s.
	(200 mg/kg)	46.10 ± 1.10**
	(300 mg/kg)	43.43 ± 1.53***

*Note:* Values were shown as mean ± SEM. (*n* = 5). One‐way ANOVA followed by Bonferroni posttest was applied, and data were represented as significant values as *p* values. n.s; not significant and *p* > 0.05, *p < 0.05, **p < 0.01, ***p < 0.001.

## 4 Discussion

Literature shows that species in genus Cornus possess therapeutic potential against various ailments. Traditionally, different parts of *C. macrophylla* were used as a potential medicinal plant for treatment of various diseases. The bark of this plant is commonly utilized in the form of powder for treatment of stomach ulcer, jaundice, and backache (Khan et al. 2009). *C. macrophylla* inhibits the activity of aldose reductase and hence can be a potent candidate for treating diabetic retinopathy (Prakash et al. 2017). Previously, diverse bioactivities such as the antifungal (Davidson and Hird 1964), antibacterial (Misra and Fridovich 1976), and antioxidant (Ohkawa et al. 1979) properties of an array of phytoconstituents isolated from Cornus species have also been investigated. In the current investigation, we explored the antioxidant capacity of using H_2_O_2_ and FRAP assays for the first time. In this assay, the DCM and EA fractions showed excellent inhibition, as shown in Tables 1 and 2. Furthermore, *Cornus mas* *s* (*C. mass*) fruit extract oral administration for 14 days to rats showed hepatoprotection by decreasing serum enzyme levels, albumin, total serum protein, and lipid peroxidation content (Alavian et al. 2014). At all concentrations, it was clearly shown that aqueous extract of *Cornus mas* *s* L. has a significant antioxidant effect on various in vitro antioxidant assays (Gülçin et al. 2005). *C. mass* (Cornelian cherry) is a valuable source of substances with a high antioxidant activity (Gąstoł et al. 2013). *C. mass* L. various genotypes showed highest antioxidant activity as genotype C24 82.37%, followed by genotype C27 77.6% and genotype C15 76.32% (Hassanpour et al. 2011). *C. mass* leaf and fruit extracts showed significant radical scavenging activity (Milenković‐Anđelković et al. 2015). In murine RAW 264.7 macrophage cells, aqueous extract of *Corni fructus* inhibited the synthesis of PGE (2) and production of NO through suppression of LPS‐induced upregulation of iNOS (inducible NO synthase) and COX‐2 (cyclooxygenase‐2) (Sung et al. 2009). *Cornus walteri* extracts have in vivo and in vitro activity significantly more sensitive to lipopolysaccharide‐induced lethality and inflammatory cytokines (Lee et al. 2011). Water and ethanolic extracts of *Cornus walteri* show potential antioxidant activity due to the presence of polyphenol and flavonoid contents (Kim et al. 2013). *C. walteri* hot water extract showed a significantly protective effect against tert‐butyl hydroperoxide‐induced oxidative stress in HepG2 cells, and also antioxidant enzymes modulation by *C. walteri* extract has important antioxidant activity against tert‐butyl hydroperoxide‐induced oxidative insult in HepG2 cells (Yeon et al. 2013). *C. walteri* ethanolic leaf extracts showed in vitro chemical and cellular antioxidant activity by treating with enzymes in human dermal fibroblast irradiated by type B ultraviolet (UVB) phototherapy (Park et al. 2014). *C. walteri* stem bark extracts showed a positive effect in lipid‐lowering, anti‐inflammatory, and antioxidative effects in rats fed a high‐fat diet (Park and Cha 2009). Fractions of ethyl acetate and aqueous from adventitious roots of *C. capitata* showed antioxidant activity against 1,1‐diphenyl‐2‐pycrylhydrazyl (DPPH) and superoxide anion radicals (Tanaka et al. 2003). *Cornus kousa* was evaluated for its antioxidant, antidiabetic, and tyrosinase inhibitory activity and exhibited a significant inhibitory effect (Lee et al. 2015). *Cornus alba* , locally known as Siberian dogwood, possesses marked anti‐inflammatory effect (Park et al. 2019). *C. macrophylla*, locally khadang, showed marked antioxidant activity (Shah et al. 2015).

In our previous investigation regarding the phytochemical investigation of *C. macrophylla* extract, results showed the occurrence of terpenes, flavonoids, alkaloids, and tannins. The cache of phytochemicals present in the extract of *C. macrophylla* may be responsible for various bioactivities. Diverse nutraceutical properties, such as anti‐inflammatory and analgesic characteristics, are associated with the presence of diverse flavonoids in the extracts (Aebi 1984; Ohkawa et al. 1979; Reddy and Kulkarni 1998). Evidently, flavonoids may have a significant effect on inflammatory biomarkers (Alavian et al. 2014). Likewise, terpenes have also been shown to possess analgesic and anti‐inflammatory activities (Gąstoł et al. 2013; Gülçin et al. 2005). These activities are ascribed to the inhibitory potential against phospholipase A2 and hence result in the obstruction of arachidonic acid metabolism (Hassanpour et al. 2011). Moreover, the presence of alkaloids is also thought to be a reason for preventing inflammation via their inhibitory effects on arachidonic acid metabolism (Milenković‐Anđelković et al. 2015; Sung et al. 2009).

The most commonly used model to assess the anti‐inflammatory potential of natural compounds in animal models is the carrageenan‐induced inflammatory mouse model (Park et al. 2019). In this model, induced inflammation occurs in two phases. In the first phase, the release of kinins, serotonin, and histamine is observed in the first hour, whereas, in 2nd phase, lysosomal enzymes and prostaglandins are released in the next 2–4 h (Gad 2018). The 2nd phase is known to be the most sensitive to anti‐inflammatory compounds and/or drugs (Ong et al. 2007). Outcomes of this study show that the administered extracts and fractions had significant inhibitory effects on carrageenan‐induced acute inflammation in the 3rd hour of the study, when compared with the standard anti‐inflammatory drug, as shown in Table 5. In the anti‐denaturation assay, heat treatment results in the denaturation of egg albumin. Likewise, native proteins and heat‐induced denatured proteins are also considered to effectively delay hypersensitivity. Furthermore, it has already been established that NSAIDs like indomethacin and phenylbutazone not only inhibit the production of endogenous prostaglandins through suppression of the COX enzyme but also through prevention of denatured proteins (Ullah et al. 2014). The current study showed that the crude extract and fractions possessed substantial anti‐inflammatory properties. Both crude extract and fractions controlled autoantigen production and hence inhibited protein denaturation as compared to the standard drug. Moreover, the secondary constituents, including tannins, flavonoids, and phenolics that were identified in our previous study, may also be responsible for this anti‐inflammatory characteristic (Khan et al. 2022).

## 5 Conclusion

It was concluded from our results that the in vivo anti‐inflammatory activity EA fraction showed strong inhibition of paw edema, while in LPS‐induced neuroprotective effect, the EA fraction showed the most potent antioxidant activity by increasing antioxidant level of GSH, SOD, and CAT in brain tissues (Tables 6, 7 and 9) and decreasing the level of MDA (Table 8). Moreover, the body weight of all the mice decreased when treated with LPS, while it increased when treated with aspirin, crude extract, DCM, and EA, respectively. Rectal temperature increased when treated with LPS and gradually decreased with aspirin, crude, DCM, and EA fraction. More research is required to evaluate the specific compounds present in *C. macrophylla* that are responsible for its neuroprotective effect.

## Author Contributions

**Ali Khan:** conceptualization (lead), data curation (lead), formal analysis (lead), investigation (lead), methodology (equal), supervision (equal), writing – original draft (lead), writing – review and editing (equal). **Aini Pervaiz:** conceptualization (supporting), data curation (supporting), investigation (lead), methodology (supporting), validation (supporting), visualization (equal), writing – original draft (equal), writing – review and editing (equal). **Muhammad Saeed Jan:** data curation (equal), investigation (supporting), methodology (supporting), visualization (supporting), writing – original draft (supporting), writing – review and editing (equal). **Bushra Ansari:** data curation (supporting), formal analysis (supporting), investigation (supporting), methodology (equal), writing – original draft (equal), writing – review and editing (equal). **Imad Ahmad:** formal analysis (supporting), investigation (supporting), methodology (supporting), validation (supporting), visualization (supporting), writing – original draft (equal), writing – review and editing (equal). **Syed Muhammad Mukarram Shah:** formal analysis (equal), investigation (supporting), methodology (supporting), validation (supporting), visualization (equal), writing – original draft (supporting), writing – review and editing (supporting). **Abdur Rauf:** conceptualization (lead), investigation (lead), methodology (lead), project administration (equal), validation (lead), visualization (supporting), writing – original draft (equal), writing – review and editing (equal). **Ahood Khalid:** methodology (supporting), validation (supporting), visualization (equal), writing – original draft (supporting), writing – review and editing (equal). **Anees Ahmed Khalil:** data curation (lead), data curation (lead), investigation (lead), methodology (equal), validation (lead), visualization (equal), writing – original draft (lead), writing – review and editing (lead). **Hassan A. Hemeg:** formal analysis (supporting), investigation (supporting), methodology (supporting), validation (equal), visualization (equal), writing – original draft (supporting), writing – review and editing (supporting). **Yahya S. Al‐Awthan:** investigation (supporting), methodology (supporting), validation (equal), visualization (equal), writing – original draft (supporting), writing – review and editing (supporting). **Omar S. Bahattab:** investigation (supporting), methodology (supporting), validation (equal), visualization (equal), writing – original draft (supporting), writing – review and editing (supporting). **Mohammed Mansour Quradha:** conceptualization (lead), data curation (supporting), investigation (supporting), methodology (lead), validation (equal), visualization (equal), writing – original draft (lead), writing – review and editing (lead).

## Conflicts of Interest

The authors declare no conflicts of interest.

## Data Availability

The data associated with this paper are given in the main text of this manuscript.

## References

[fsn370620-bib-0001] Aebi, H. 1984. “Catalase In Vitro.” In Methods in Enzymology, vol. 105, 121–126. Academic Press. 6727660 10.1016/s0076-6879(84)05016-3

[fsn370620-bib-0002] Agarwal, H. , and V. K. Shanmugam . 2022. “Mechanism‐Based Approaches to Medicinal Plant Mediated Treatment of Inflammatory Disorders: A Review.” South African Journal of Botany 147: 380–390.

[fsn370620-bib-0003] Alavian, S. M. , N. Banihabib , M. E. Haghi , and F. Panahi . 2014. “Protective Effect of *Cornus mas* Fruits Extract on Serum Biomarkers in CCl4‐Induced Hepatotoxicity in Male Rats.” Hepatitis Monthly 14, no. 4: e10330. 24829584 10.5812/hepatmon.10330 PMC4006099

[fsn370620-bib-0004] Anjum, N. A. , S. Umar , and M. T. Chan , eds. 2010. Ascorbate‐Glutathione Pathway and Stress Tolerance in Plants. Springer Science & Business Media.

[fsn370620-bib-0005] Ayala, A. , M. F. Muñoz , and S. Argüelles . 2014. “Lipid Peroxidation: Production, Metabolism, and Signaling Mechanisms of Malondialdehyde and 4‐Hydroxy‐2‐Nonenal.” Oxidative Medicine and Cellular Longevity 2014, no. 1: 360438. 24999379 10.1155/2014/360438 PMC4066722

[fsn370620-bib-0006] Calder, P. C. , N. Bosco , R. Bourdet‐Sicard , et al. 2017. “Health Relevance of the Modification of Low Grade Inflammation in Ageing (Inflammageing) and the Role of Nutrition.” Ageing Research Reviews 40: 95–119. 28899766 10.1016/j.arr.2017.09.001

[fsn370620-bib-0007] Davidson, B. E. , and F. J. R. Hird . 1964. “The Estimation of Glutathione in Rat Tissues. A Comparison of a New Spectrophotometric Method With the Glyoxalase Method.” Biochemical Journal 93, no. 2: 232–236. 5891310 10.1042/bj0930232 PMC1206282

[fsn370620-bib-0009] Du, Z. , J. Liu , D. Zhang , et al. 2019. “Individual and Synergistic Antioxidant Effects of Dipeptides in In Vitro Antioxidant Evaluation Systems.” International Journal of Peptide Research and Therapeutics 25: 391–399.

[fsn370620-bib-0010] Estakhr, J. , and N. Javdan . 2011. “Spermatogenic Activity of *Aloe Vera* in Adult Male Rats.” Pharmacology 2: 886–889.

[fsn370620-bib-0011] Gad, S. 2018. “Effect of Ginger as Anti‐Inflammatory Agent on Serum Nitric Oxide, Tumor Necrotic Factor α (TNF‐α) and Interleukin 4 (IL‐4) in Albino Rats With Carrageenan Induced Paw Edema.” Virology & Immunology Journal 2, no. 8: 000179.

[fsn370620-bib-0012] Gąstoł, M. , M. Krośniak , M. Derwisz , and J. Dobrowolska‐Iwanek . 2013. “Cornelian Cherry ( *Cornus mas* L.) Juice as a Potential Source of Biological Compounds.” Journal of Medicinal Food 16, no. 8: 728–732. 23905648 10.1089/jmf.2012.0248

[fsn370620-bib-0013] Gaweł, S. , M. Wardas , E. Niedworok , and P. Wardas . 2004. “Malondialdehyde (MDA) as a Lipid Peroxidation Marker.” Wiadomości Lekarskie 57, no. 9–10: 453–455. 15765761

[fsn370620-bib-0014] Gülçin, İ. , Ş. Ü. K. R. Ü. Beydemir , G. Şat , and Ö. İ. Küfrevioğlu . 2005. “Evaluation of Antioxidant Activity of Cornelian Cherry (*Cornus mas* L.).” Acta Alimentaria 34, no. 2: 193–202.

[fsn370620-bib-0015] Hassanpour, H. , H. Yousef , H. Jafar , and A. Mohammad . 2011. “Antioxidant Capacity and Phytochemical Properties of Cornelian Cherry ( *Cornus mas* L.) Genotypes in Iran.” Scientia Horticulturae 129, no. 3: 459–463.

[fsn370620-bib-0016] Jan, M. S. , S. Ahmad , F. Hussain , et al. 2020. “Design, Synthesis, In‐Vitro, In‐Vivo and In‐Silico Studies of Pyrrolidine‐2, 5‐Dione Derivatives as Multitarget Anti‐Inflammatory Agents.” European Journal of Medicinal Chemistry 186: 111863. 31740050 10.1016/j.ejmech.2019.111863

[fsn370620-bib-0017] Kaushal, J. , S. Mehandia , G. Singh , A. Raina , and S. K. Arya . 2018. “Catalase Enzyme: Application in Bioremediation and Food Industry.” Biocatalysis and Agricultural Biotechnology 16: 192–199.

[fsn370620-bib-0018] Khan, A. , A. Pervaiz , B. Ansari , et al. 2022. “Phytochemical Profiling, Anti‐Inflammatory, Anti‐Oxidant and In‐Silico Approach of *Cornus Macrophylla* Bioss (Bark).” Molecules 27, no. 13: 4081. 35807324 10.3390/molecules27134081 PMC9268425

[fsn370620-bib-0019] Khan, I. , M. Nisar , F. Ebad , et al. 2009. “Anti‐Inflammatory Activities of Sieboldogenin From *Smilax china* Linn.: Experimental and Computational Studies.” Journal of Ethnopharmacology 121, no. 1: 175–177. 19007873 10.1016/j.jep.2008.10.009

[fsn370620-bib-0020] Kim, Y. S. , J. W. Hwang , S. H. Kang , et al. 2013. “Protective Effects of Cornus Walteri W. Extracts on t‐BHP‐Induced Cell Damage Through Antioxidant Activity.” Biotechnology and Bioprocess Engineering 18: 819–826.

[fsn370620-bib-0021] Landis, G. N. , and J. Tower . 2005. “Superoxide Dismutase Evolution and Life Span Regulation.” Mechanisms of Ageing and Development 126, no. 3: 365–379. 15664623 10.1016/j.mad.2004.08.012

[fsn370620-bib-0022] Lee, E. H. , S. H. Lee , and Y. J. Cho . 2015. “Biological Activities of Extracts From *Cornus Kousa* Fruit.” Journal of Applied Biological Chemistry 58, no. 4: 317–323.

[fsn370620-bib-0023] Lee, S. H. , K. R. Yoon , E. Lee , and Y. Y. Cha . 2011. “Anti‐Inflammatory Effect of Cornus Walteri.” Journal of Physiology & Pathology in Korean Medicine 25, no. 6: 982–988.

[fsn370620-bib-0024] Lesser, M. P. 2005. “Oxidative Stress in Marine Environments: Biochemistry and Physiological Ecology.” Annual Review of Physiology 68: 253–278. 10.1146/annurev.physiol.68.040104.110001 16460273

[fsn370620-bib-0025] Milenković‐Anđelković, A. S. , M. Z. Anđelković , A. N. Radovanović , B. C. Radovanović , and V. Nikolić . 2015. “Phenol Composition, DPPH Radical Scavenging and Antimicrobial Activity of Cornelian Cherry (*Cornus mas*) Fruit and Leaf Extracts.” Hemijska Industrija 69, no. 4: 331–337.

[fsn370620-bib-0026] Misra, H. P. , and I. Fridovich . 1976. “The Oxidation of Phenylhydrazine: Superoxide and Mechanism.” Biochemistry 15, no. 3: 681–687. 175827 10.1021/bi00648a036

[fsn370620-bib-0027] Ngo, Y. L. , C. H. Lau , and L. S. Chua . 2018. “Review on Rosmarinic Acid Extraction, Fractionation and Its Anti‐Diabetic Potential.” Food and Chemical Toxicology 121: 687–700. 30273632 10.1016/j.fct.2018.09.064

[fsn370620-bib-0028] Noshiro, S. , and P. Baas . 2000. “Latitudinal Trends in Wood Anatomy Within Species and Genera: Case Study in Cornus Sl (Cornaceae).” American Journal of Botany 87, no. 10: 1495–1506. 11034925

[fsn370620-bib-0029] Ohkawa, H. , N. Ohishi , and K. Yagi . 1979. “Assay for Lipid Peroxides in Animal Tissues by Thiobarbituric Acid Reaction.” Analytical Biochemistry 95, no. 2: 351–358. 36810 10.1016/0003-2697(79)90738-3

[fsn370620-bib-0030] Ong, C. K. S. , P. Lirk , C. H. Tan , and R. A. Seymour . 2007. “An Evidence‐Based Update on Nonsteroidal Anti‐Inflammatory Drugs.” Clinical Medicine & Research 5, no. 1: 19–34. 17456832 10.3121/cmr.2007.698 PMC1855338

[fsn370620-bib-0031] Pan, M. H. , C. S. Lai , and C. T. Ho . 2010. “Anti‐Inflammatory Activity of Natural Dietary Flavonoids.” Food & Function 1, no. 1: 15–31. 21776454 10.1039/c0fo00103a

[fsn370620-bib-0032] Pan, S. Y. , G. Litscher , S. H. Gao , et al. 2014. “Historical Perspective of Traditional Indigenous Medical Practices: The Current Renaissance and Conservation of Herbal Resources.” Evidence‐Based Complementary and Alternative Medicine 2014, no. 1: 525340. 24872833 10.1155/2014/525340 PMC4020364

[fsn370620-bib-0033] Park, D. H. , J. Yin , H. S. Wang , M. Kim , M. J. Kim , and M. W. Lee . 2019. “Validation and Content Analysis of Cornusiin B Isolated From *Cornus alba* .” Korean Journal of Pharmacognosy 50, no. 3: 155–158.

[fsn370620-bib-0034] Park, H. C. , T. K. Jung , and K. S. Yoon . 2014. “Antioxidative Activity of Extract of Cornus Walteri Wanger Leaves in Human Dermal Fibroblast Irradiated by UVB.” Korean Society for Biotechnology and Bioengineering Journal 29, no. 6: 432–436.

[fsn370620-bib-0035] Park, W. H. , and Y. Y. Cha . 2009. “Effects of Stem Bark Extracts of Cornus Walteri Wanger on the Lipid Lowering, Anti‐Oxidative Activity and Concentration of Proinflammatory Cytokines in Rat Fed High Fat Diet.” Journal of Korean Medicine Rehabilitation 19, no. 4: 59–78.

[fsn370620-bib-0036] Prakash, R. , E. Sandhya , N. Ramya , R. Dhivya , M. Priyadarshini , and B. Sakthi Priya . 2017. “Neuroprotective Activity of Ethanolic Extract of Tinospora Cordifolia on LPS Induced Neuroinflammation.” Translational Biomedicine 8, no. 4: 135.

[fsn370620-bib-0037] Reddy, D. S. , and S. K. Kulkarni . 1998. “Possible Role of Nitric Oxide in the Nootropic and Antiamnesic Effects of Neurosteroids on Aging‐and Dizocilpine‐Induced Learning Impairment.” Brain Research 799, no. 2: 215–229. 9675286 10.1016/s0006-8993(98)00419-3

[fsn370620-bib-0038] Ruch, R. J. , S. J. Cheng , and J. E. Klaunig . 1989. “Prevention of Cytotoxicity and Inhibition of Intercellular Communication by Antioxidant Catechins Isolated From Chinese Green Tea.” Carcinogenesis 10, no. 6: 1003–1008. 2470525 10.1093/carcin/10.6.1003

[fsn370620-bib-0039] Sadiq, A. , A. Zeb , S. Ahmad , et al. 2019. “Evaluation of Crude Saponins, Methanolic Extract and Subsequent Fractions From Isodon Rugosus Wall. Ex Benth: Potentials of Anti‐Angiogenesis in Egg and Anti‐Tumorigenesis in Potato.” Pakistan Journal of Pharmaceutical Sciences 32, no. 5: 1971–1977. 31813860

[fsn370620-bib-0040] Shah, S. , S. M. M. Shah , Z. Ahmad , et al. 2015. “Phytochemicals, In Vitro Antioxidant, Total Phenolic Contents and Phytotoxic Activity of *Cornus macrophylla* Wall Bark Collected From the North‐West of Pakistan.” Pakistan Journal of Pharmaceutical Sciences 28, no. 1: 23–28. 25553682

[fsn370620-bib-0041] Sung, Y. H. , H. K. Chang , S. E. Kim , et al. 2009. “Anti‐Inflammatory and Analgesic Effects of the Aqueous Extract of Corni Fructus in Murine RAW 264.7 Macrophage Cells.” Journal of Medicinal Food 12, no. 4: 788–795. 19735178 10.1089/jmf.2008.1011

[fsn370620-bib-0042] Tanaka, N. , K. Nishikawa , and K. Ishimaru . 2003. “Antioxidative Capacity of Extracts and Constituents in *Cornus capitata* Adventitious Roots.” Journal of Agricultural and Food Chemistry 51, no. 20: 5906–5910. 13129293 10.1021/jf030267s

[fsn370620-bib-0043] Tasneem, S. , B. Liu , B. Li , M. I. Choudhary , and W. Wang . 2019. “Molecular Pharmacology of Inflammation: Medicinal Plants as Anti‐Inflammatory Agents.” Pharmacological Research 139: 126–140. 30395947 10.1016/j.phrs.2018.11.001

[fsn370620-bib-0044] Trivedi, D. K. , S. S. Gill , S. Yadav , and N. Tuteja . 2013. “Genome‐Wide Analysis of Glutathione Reductase (GR) Genes From Rice and Arabidopsis.” Plant Signaling & Behavior 8, no. 2: e23021. 23221779 10.4161/psb.23021 PMC3657001

[fsn370620-bib-0045] Tsikas, D. 2017. “Assessment of Lipid Peroxidation by Measuring Malondialdehyde (MDA) and Relatives in Biological Samples: Analytical and Biological Challenges.” Analytical Biochemistry 524: 13–30. 27789233 10.1016/j.ab.2016.10.021

[fsn370620-bib-0046] Ullah, H. A. , S. Zaman , F. Juhara , et al. 2014. “Evaluation of Antinociceptive, In‐Vivo & In‐Vitro Anti‐Inflammatory Activity of Ethanolic Extract of *Curcuma zedoaria* Rhizome.” BMC Complementary and Alternative Medicine 14: 1–12. 25242194 10.1186/1472-6882-14-346 PMC4190444

[fsn370620-bib-0047] Vasiliev, V. V. , V. A. Barynin , and A. F. Rasin . 2001. “Anisogrid Lattice Structures–Survey of Development and Application.” Composite Structures 54, no. 2–3: 361–370.

[fsn370620-bib-0048] Wencel‐Delord, J. , M. Mauduit , and C. Crévisy . 2001. Encyclopedia of Reagents for Organic Synthesis. Wiley.

[fsn370620-bib-0049] Wu, Y. R. , C. H. Hsing , C. J. Chiu , H. Y. Huang , and Y. H. Hsu . 2022. “Roles of IL‐1 and IL‐10 Family Cytokines in the Progression of Systemic Lupus Erythematosus: Friends or Foes?” IUBMB Life 74, no. 2: 143–156. 34668305 10.1002/iub.2568

[fsn370620-bib-0050] Yeon, S. H. , H. Ham , J. Sung , et al. 2013. “Antioxidant Activities of Hot Water Extract From Cornus Walteri Wanger Against Oxidative Stress Induced by Tert‐Butyl Hydroperoxide in HepG2 Cells.” Journal of the Korean Society of Food Science and Nutrition 42, no. 10: 1525–1532.

